# Formaline as a Preserving and Hardening Fluid for Histological Purposes

**Published:** 1895-09

**Authors:** 


### Formaline as a Preserving and Hardening Fluid for Histological Purposes.

G. Bergonzoli (*Bull. Scientifico*, 1894, No. 1, p. 18) says formaline or formal, in solution, concentrated to 40 per cent. of formaldehyde, is a limpid liquid, slightly opalescent, neutral or slightly acid, of a characteristic pungent odor. The antiseptic properties of formaldehyde have been studied by Loew (1886), Aronson, Berlioz, and Trillat. The author has found from his observation that solutions of formaline are deodorant and disinfectant; that pieces of tissue immersed in it are rapidly fixed and hardened, and only shrink to an almost imperceptible degree. The color is perfectly preserved, only the coloring matter of the blood being dissolved. For nervous tissue it is excellent. Formaline has the advantage over alcohol that it is not inflammable and is much cheaper.—*Rev. Internat. de Biblog. Med.*

